# Relationship between VEGF Gene Polymorphisms and Serum VEGF Protein Levels in Patients with Rheumatoid Arthritis

**DOI:** 10.1371/journal.pone.0160769

**Published:** 2016-08-11

**Authors:** Agnieszka Paradowska-Gorycka, Andrzej Pawlik, Katarzyna Romanowska-Prochnicka, Ewa Haladyj, Damian Malinowski, Barbara Stypinska, Malgorzata Manczak, Marzena Olesinska

**Affiliations:** 1 Department of Biochemistry and Molecular Biology, National Institute of Geriatrics, Rheumatology and Rehabilitation, Warsaw, Poland; 2 Department of Physiology, Pomeranian Medical University, Szczecin, Poland; 3 Department of Connective Tissue Diseases, National Institute of Geriatrics, Rheumatology and Rehabilitation, Warsaw, Poland; 4 Department of Pathophysiology, Warsaw Medical University, Warsaw, Poland; 5 Department of Pharmacology, Pomeranian Medical University, Szczecin, Poland; 6 Department of Gerontology and Public Health, National Institute of Geriatrics, Rheumatology and Rehabilitation, Warsaw, Poland; Universite de Nantes, FRANCE

## Abstract

**Background:**

Rheumatoid arthritis (RA) is one of the chronic autoimmune diseases, with genetic and environmental predisposition, and synovial angiogenesis is considered to be a notable stage in its pathogenesis. Angiogenesis or vascular proliferation has been suggested to be a pivotal mechanism involved in both inflammation/immune activation and joint invasion and destruction. RA may be considered an “angiogenic disease” because it is associated with active tissue neovascularization. Vascular endothelial growth factor (VEGF) promotes vascular permeability, regulates angiogenesis, endothelial cell proliferation and migration, chemotaxis, and capillary hyper permeability and therefore is involved in the development of inflammation. VEGF is the most potent proangiogenic molecule promoting the angiogenic phenotype of RA and is upregulated in RA.

**Objectives:**

The aim of the study was to identify functional VEGF variants and their possible association with VEGF expression, susceptibility to and severity of RA.

**Methods:**

581 RA patients and of 341 healthy individuals were examined for -1154 A/G, -2578 A/C VEGF gene polymorphisms by PCR-RFLP method and for -634 G/C VEGF gene polymorphisms by TaqMan SNP genotyping assay. Serum VEGF levels in RA patients and controls were measured by ELISA.

**Results:**

The -1154 A/G VEGF gene polymorphism under the codominant, recessive (AA+AG vs. GG) and dominant (AA vs. AG+GG) models were associated with RA (p = 0.0009; p = 0.004; p = 0.017, respectively). VEGF -2578 A/C revealed differences in the case-control distribution in codominant, recessive, dominant and overdominant models (all p<0.0001). Furthermore, the -634 G/C VEGF gene SNP was not correlated with susceptibility to RA in Polish population. The genotype-phenotype analysis showed significant association between the VEGF -1154 A/G and -634 G/C and mean value of the hemoglobin (all p = 0.05), additionally they relevated that the number of women with the polymorphic allele -2578 C was lower than the number of women with wild type allele -2578A (p = 0.006). Serum VEGF levels were significantly higher in RA patients than in control groups (both p = 0,0001).

**Conclusion:**

Present findings indicated that VEGF genetic polymorphism as well as VEGF protein levels may be associated with the susceptibility to RA in the Polish population.

## Introduction

Rheumatoid arthritis (RA) is an immune-inflammatory disease characterized by progressively destructive joint inflammation, destruction of articular cartilage and synovialhyperplasia, without precisely known pathogenesis. Heterogeneous phenotypes of RA suggested that both environmental and genetic factors contribute to the susceptibility of RA as reflected by familial clustering. The total heritability of RA has been estimates about 66% [1]. In particular, genome-wide association and case-control studies in a large number of RA patients have significantly expanded our understanding of the genetic basis of RA. In addition to being an inflammatory condition, RA is also considered to be a member of the “angiogenic family of diseases” because it is connected with tissue neovascularization [2, 3]. Neovascularization, or angiogenesis, is a complex process leading to formation of new blood vessels from the pre-existing vascular network of the tissue. Synovial angiogenesis may plays a critical role in the early stage of RA by promoting inflammatory cell infiltration and the development of pannus, aggressive tumor like fibrovascular granulation tissue, which eventually leads to joint destruction [4–7]. However, the molecular mechanisms, which participate in the promotion of the angiogenesis in rheumatoid arthritis, have not been identified [8]. Moreover, inflammation is often accompanied by imbalanced angiogenesis, and vascular endothelial growth factor (VEGF) is considered to provide a linkage between these processes [9, 10, 11].

VEGF is one of the most potent proangiogenic factors, which expression is potentiated in response to the hypoxic state in the rheumatoid joints and by several of pro- and anti-inflammatory cytokines [5, 12]. It stimulates angiogenesis by promoting of endothelial cells proliferation and migration to form new blood vessels and increase vascular permeability as well as it induces several of proinflammatory changes in chronic inflammation [13–15]. Although the role of VEGF in joint inflammation is known, it role in joint destruction is not well understood [11]. Moreover, VEGF stimulates pannus formation, and as the pannus grows, more VEGF is produced, forming a vicious circle. In patients with RA, VEGF and its receptor have been shown to be expressed in the synovial tissue of inflamed joints [5, 16, 17]. The VEGF expression increased in both serum and synovial fluid of rheumatoid arthritis patients and it was associated with disease activity, inflammatory markers, destructive changes, and pathological features of arthritis as well as angiogenesis [17–21]. High levels of this mitogen may be also associated with an accelerated atherosclerosis and increased risk of cardiovascular disease (CVD) in patients with RA, which suggested that increased level of VEGF may be potential marker for patients with increased risk of severe, life-threatening complication.

In the present study, based on the hypothesis of a close relationship between inflammation and atherosclerosis, we have tested whether genetic variants in *VEGF* gene are associated with RA in a predominantly Polish population using a case-control approach. In a subgroup of 385 patients we compared VEGF protein levels with severity of RA and in relation to *VEGF* genotypes. Finally, we investigated whether *VEGF* gene variants and circulating level of VEGF are related to the development of CVD in our RA patients.

## Materials and Methods

### Study population

A study group consisted of 564 patients with established RA and 341 unrelated healthy controls without history of immunological diseases. All the patients included on the study were of European descent and had been diagnosed with RA according to the 1987 classification criteria of the American College of rheumatology (ACR). Patients were recruited from the Connective Tissue Diseases Department of the Institute of Rheumatology in Warsaw and from the Pomeranian Medical University, Szczecin, Poland. The control groups (217 females and 124 males, age between 18 and 63 years) were selected randomly from blood bank donors from healthy volunteers who had no a history of autoimmune diseases. Patients and control subjects had the same ethnicity, socioeconomic status and were from the same geographical area. We selected a representative sample of the admixed urban Polish population.

Our study was approved by the Research Ethics Committee of the National Institute of geriatrics, Rheumatology and Rehabilitation in Warsaw and of the Pomeranian Medical University in Szczecin. All participants, RA patients and healthy subjects, provided written informed consent according to the Declaration of Helsinki as revised in 2000.

#### Single nucleotide polymorphisms (SNPs) selection and genotyping

The three *VEGF* SNPs studied -1154 A/G (rs1570360), -2578 A/C (rs699947) and -634 G/C (rs2010963) were selected from previous study in rheumatic diseases [5, 13, 22]. Genomic DNA was isolated from whole blood collected in EDTA tubes from patients with RA and the control group using the standard isothiocyanate guanidine extraction method and/or the QIAamp DNA Blood Mini Kit (Qiagen). DNA purity and concentration were determined by spectrophotometric measurement of absorbance at 260 and 280 nm.

Genotyping of -634 G/C (rs2010963) was analyzed using TaqMan SNP Genotyping Assays (C___8311614_10; Applied Biosystems, Forester City, CA, USA). The reaction was performed in 10 ul volumes on StepOne real-time PCR system following the manufacturer’s protocol. Allelic discrimination was conducted in a Rotor-Gene 6000 Real-Timer PCR system (Corbett Research).

Furthermore, genotyping of -1154 A/G (rs1570360) and -2578 A/C (rs699947) were determined using the polymerase chain reaction (PCR)-restriction fragment length polymorphism (RFLP) method was performed using the following primers: **-1154 A/G (rs1570360)** forward 5”–CGC GTG TCT CTG GAC AGA GTT TCC– 3’ and reverse 5’- CGG GGA CAG GCC AGC TTC AG– 3’, to generated a 173 bp product; **-2578 A/C (rs699947)** forward 5’–GGC CTT AGG ACA CCT ACC– 3’ and reverse 5’–CAC AGC TTC TCC CCT ATA C– 3’, to generate a 456 bp product. Amplification reaction was performed with 200 ng of genomic DNA in a 50-μl PCR mixture using 10 pmol of each primer, 0·25 mM each deoxyribonucleoside triphosphate (dNTP) (Qiagen), 1 U HotStar Taq polymerase (Qiagen) and ×1 PCR buffer (containing 1·5 μM magnesium chloride; Qiagen). 10 μl of PCR product was digested with 1 μl MnlI (EURx, Gdansk, Poland) for the -1154 A/G (rs1570360) and with the 1 ul BglII (EURx, Gdansk, Poland) for the -2578 A/C (rs699947). All restriction digest were performed at 37°C for 1 hour.

The genotypes obtained in this study were subsequently validated and confirmed by sequencing the PCR products using an ABI PRISM Sequencer (Applied Biosystems).

### Assay for serum levels of VEGF

Serum samples were separated from peripheral venous blood and collected at −86°C until analysis. VEGF levels were determined using a human VEGF quantitative enzyme-linked immunosorbent assay (ELISA; R&D systems, Minneapolis, MN, USA), according to the manufacture instructions. The minimum level of detection for VEGF was 9 pg/ml. All analysis was run in duplicate and the intra-assay coefficient of variation was < 10%. The plates were read using an ELISA reader (El × 800, BIO-TEK Instruments, Winooski, VT, USA) at 450 nm.

### Statistical analysis

Comparison of genotype distribution and allele frequencies between RA patients and the control group were estimated by computing odds ratios (ORs) and 95% confidence intervals (CIs). The association between target SNPs and the risk of RA was analysed by unconditional logistic regression using four genetic models, including co-dominant, dominant, recessive and over-dominant models evaluated using the χ2 test. For genetic association analyses, all polymorphisms were tested for deviations from the Hardy–Weinberg equilibrium (HWE) using the HardyWeinberg Simulator software (available at Institute of Human Genetics, Helmholtz Zentrum München, Germany). Linkage disequilibrium (LD), coefficient (D′ and r2) for haplotypes and their frequencies were performed using the genetic statistical software SHEsis (http://shesisplus.bio-x.cn/SHEsis.html) [23, 24]. The association between SNPs and clinical/serological parameters was assessed by χ2 test with Yates’ correction (categorical variables) or Mann–Whitney U-test (continuous variables). A p-value of <0.05 was considered statistically significant.

## Results

### Characteristics of the study patients

Data on the main demographic data, clinical characteristics and CV events of patients enrolled in the study are shown in Table 1. The median age of the patients was 56 years; 88% were women; and 69% were rheumatoid factor (RF) positive. They had clinically active disease with DAS-28 score >3.9 and Larsen score 3. Evidence of coronary artery disease was found in 14% of patients; hypertension in 36%, and myocarditis in 3%; all this symptoms were classified as CVD. Comparison between RA patients who had a CV disease and those without CVD (Table 2) demonstrated that patients with CVD were older (62 vs 52 yrs; p<0.001) and had a higher HAQ score (1.6 vs 1.4; p = 0.015) and mean value of creatinine (p = 0.010) than those without CVD. Moreover, we also observed a tendency to higher mean value of ESR (p = 0.089) and PLT (p = 0.099) in RA patients with CVD. In contrast, RA patients without CVD had a tendency to higher frequency of being positive for RF (p = 0.099) and anti-CCP (p = 0.080). There was no significant difference in disease duration and DAS-28 score between patients with and those without CVD.

**Table 1:** Demographic and clinical characteristics of RA patients.

Characteristics	RA patients	
	N	median (IQR)
Age [years]	*541*	56 (50–65)
Disease duration [years]	*495*	10 (5–16)
Larsen	*541*	3 (3–3)
Number of tender joints	*309*	7 (3–12)
Number of swollen joints	*309*	3 (1–7)
ESR [mm/h]	*538*	30 (17–50)
CRP [mg/L]	*312*	13,1 (6,0–32,0)
Hemoglobin [g/dL]	*312*	12,7 (11,6–13,5)
VAS [mm]	*305*	52 (32–70)
DAS 28-CRP	*306*	5,0 (3,9–5,9)
PLT [x10^3^/mm^3^]	*312*	311,5 (254–383)
Creatinine	*311*	0,7 (0,6–0,8)
HAQ	*292*	1,5 (1,0–2,0)
	**N**	**n (%)**
Sex (female)	*558*	492 (88%)
RF presence	*534*	369 (69%)
anti-CCP presence	*314*	60 (19%)
Morning stiffness	*336*	262 (78%)
Coronary artery disease	*310*	42 (14%)
Hypertension	*311*	113 (36%)
Myocarditis	*308*	10 (3%)
Diabetes	*310*	15 (5%)

N—number of patients with clinical information; n- number of patients with positive clinical manifestation; IQR—interquartile range; DAS-28—disease activity score for 28 joints, VAS—visual analogue scale (range 0–100), HAQ—Health Assessment Questionnaires (range 0–3), CRP—C-reactive protein, ESR—erythrocyte sedimentation ratio, PLT—platelet, RF—rheumatoid factor (>34 IU / ml), anti-CCP—anti-CCP antibodies (>17 U/ ml)

**Table 2:** Demographic and clinical characteristics of the RA patients with CVD and without CVD.

**parameter**	**patients with CVD**		**patients without CVD**		**p**
	***N***	**median (IQR)**	***N***	**median (IQR)**	
age [years]	*134*	62 (56–68)	*177*	52 (44–59)	**< 0.001**
disease duration [years]	*122*	10 (6–17)	*145*	11 (5–17)	0.976
number of swollen joints	*131*	3 (1–7)	*173*	4 (1–8)	0.154
number of tender joints	*131*	8 (4–14)	*173*	7 (2–11)	0.336
Larsen	*133*	3 (2–4)	*177*	3 (3–4)	0.731
ESR [mm/h]	*133*	30 (15–43)	*176*	24 (12–39.5)	**0.089**
CRP [mg/L]	*130*	15.0 (7.6–36.5)	*176*	12.6 (5.0–32.0)	0.101
VAS [mm]	*131*	55 (30–75)	*170*	51 (32–67)	0.263
DAS-28	*130*	5.1 (4.0–6.0)	*172*	5.0 (3.8–5.9)	0.261
HAQ	*123*	1.6 (1.0–2.1)	*159*	1.4 (0.8–1.9)	**0.015**
Hb	*131*	12.9 (11.7–13.6)	*175*	12.5 (11.4–13.4)	0.228
PLT	*131*	301 (243–362)	*175*	323 (266–393)	**0.099**
Creatinine	*131*	0.7 (0.6–0.9)	*174*	0.7 (0.6–0.8)	**0.010**
**parameter**	**patients with CVD**		**patients without CVD**		**p**
	*N*	n (%)	*N*	n (%)	
women	*134*	124 (93%)	*177*	164 (93%)	0.969
RF +	*133*	93 (70%)	*171*	104 (61%)	**0.099**
anti-CCP +	*132*	113 (86%)	*175*	136 (78%)	**0.080**

### Association of *VEGF* SNPs with RA in a Polish population

First we explored whether there was a relationship between functional *VEGF*-1154 A/G (rs1570360), -2578 A/C (rs699947) and -634 G/C (rs2010963) variants and RA in our cohort. The distributions of allelic and genotype frequencies of the polymorphisms in *VEGF* gene among patients and controls are shown in Table 3. Genotype frequencies for the three studied SNPs were in Hardy-Weinberg equilibrium (HWE) with exception of -2578 A/C (rs699947) for the RA group (p = 0.01). Genotyping (real-time PCR and sequencing) was repeated on randomly selected samples, giving complete conformity of the results.

**Table 3:** Distribution of genotypes and allele frequencies of VEGF SNPs among Polish patients with RA and healthy subjects. OR adjusted for sex and age.

Genotype		RA n (%)	Control n (%)	adjusted OR (95% CI)	p value
**VEGF-1154 A/G**					
Codominant	AA	131 (23%)	111 (33%)	1	-
	AG	301 (54%)	177 (52%)	1.23 (0.92–1.64)	0.159
	GG	127 (24%)	53 (16%)	1.24 (0.86–1.80)	0.251
Dominant	AA	131 (23%)	111 (33%)	1	-
	AG+GG	428 (77%)	230 (67%)	1.37 (1.08–1.75)	**0.010**
Recessive	AA+AG	432 (77%)	288 (84%)	1	-
	GG	127 (23%)	53 (16%)	0.90 (0.69–1.18)	0.450
**VEGF-2578 A/C**					
Codominant	AA	164 (29%)	218 (64%)	1	-
	AC	251 (45%)	104 (30%)	0.92 (0.66–1.28)	0.631
	CC	144 (26%)	19 (6%)	3.18 (2.02–5.02)	**0.000**
Dominant	AA	164 (29%)	218 (64%)	1	**-**
	AC+CC	395 (71%)	123 (36%)	1.93 (1.55–2.41)	**0.000**
Recessive	AA+AC	415 (74%)	322 (94%)	1	**-**
	CC	144 (26%)	19 (6%)	2.44 (1.74–3.44)	**0.000**
**VEGF -634 G/C**					
Codominant	GG	296 (53%)	173 (51%)	1	-
	GC	227 (40%)	142 (42%)	1.01 (0.72–1.42)	0.943
	CC	40 (7%)	26 (8%)	1.00 (0.61–1.66)	0.997
Dominant	GG	296 (53%)	173 (51%)	1	-
	GC+CC	267 (47%)	168 (49%)	1.01 (0.82–1.25)	0.912
Recessive	GG+GC	523 (93%)	315 (92%)	1	-
	CC	40 (7%)	26 (8%)	1.00 (0.69–1.46)	0.995

p—χ^2^ test with Yate’ correction, p = RA vs controls, p≤0,05 was considered as significant

Three genetic models, including codominant, dominant and recessive were applied to assess the association of SNPs within the *VEGF* gene and RA risk. The analysis of the *VEGF* -2578 A/C (rs699947) polymorphism revealed significant differences in the case—control distribution in all examined models. In our population, the CC genotype of -2578 A/C (rs699947) was associated with RA (co-dominant: OR = 0.92; 95% CI 0.66 to1.28; recessive: OR = 2.44; 95% CI 1.74 to 3.44; dominant: OR = 1.93; 95% CI 1.55 to 2.41; all p = 0.000). With regard to the -1154 A/G (rs1570360) *VEGF* gene polymorphism, RA patients showed significantly different genotype distribution compared to control subjects in one models. Under the dominant model the frequency of the AG+GG genotype was significantly higher in patients with RA compared to the healthy subjects (77% vs 67%; p = 0.010; OR = 1.37; 95% CI 1.08 to 1.75).

No significant differences were observed in the proportion of cases and control under each genetic model for the *VEGF* -634 G/C (rs2010963) variant.

### *VEGF* haplotypes and risk of RA

**Fig 1:**
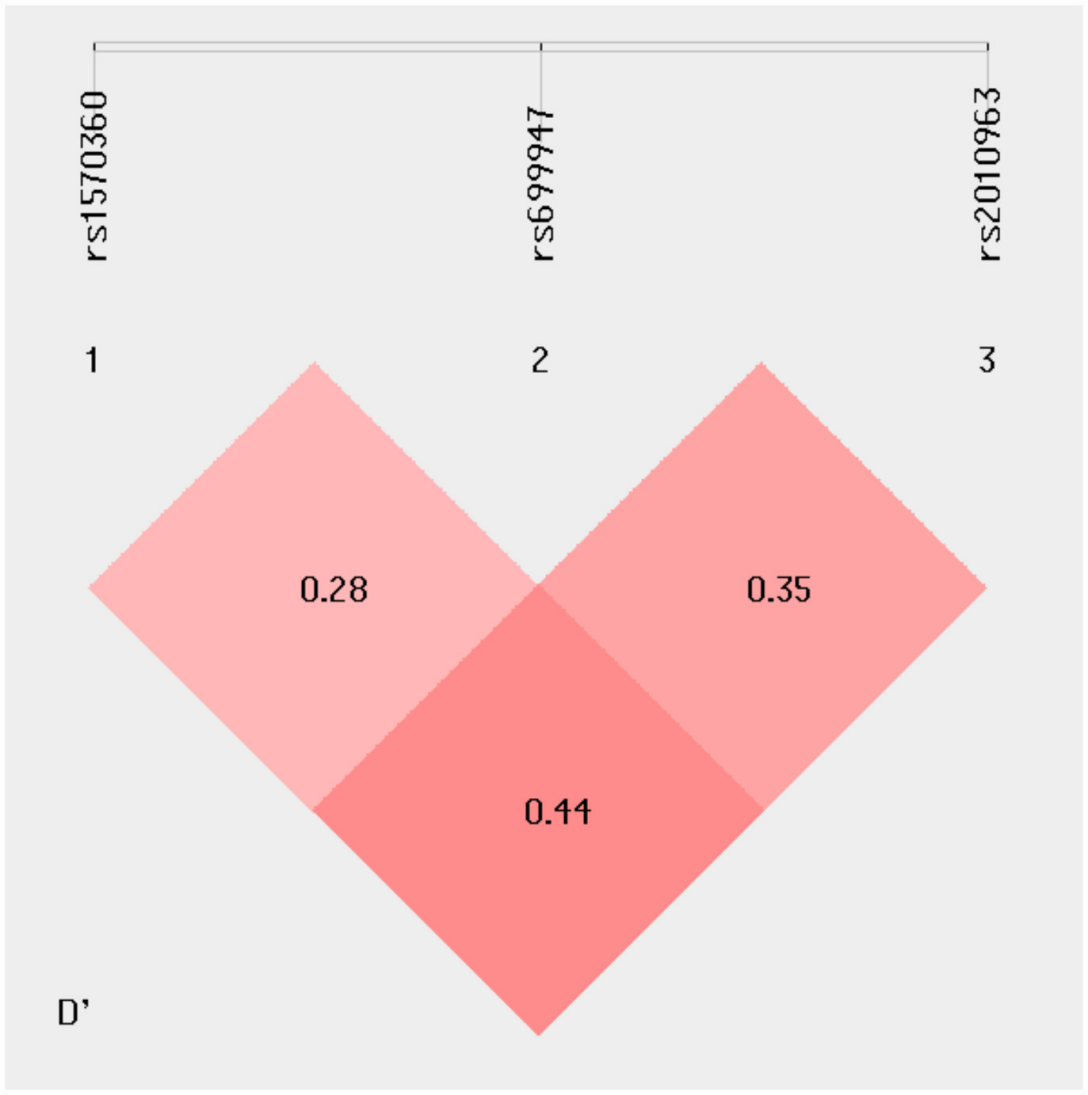
Linkage disequilibrium (LD) plots of three SNPs in the VEGF gene.

To further investigate whether haplotypes of *VEGF* were correlated with RA, the LD and haplotype frequencies differences were estimated for the 3 identified polymorphisms in the *VEGF* gene. The pattern of LD in the *VEGF* locus was measured by D’ and r^2^ score. The interaction between any possible pair of SNPs was visualized by SHEsis program (Fig 1). Analysis demonstrated that studied *VEGF* SNPs were in indistinct linkage disequilibrium (LD) with D’ = 0.28–0.44 and r^2^ = 0.03–0.06. Of the possible total of 8 haplotypes, only 7 were common (frequency >0.03 in both case and controls). Of these seven haplotypes, haplotypes GAG, AAG and AAC were negatively (all p <0.0001) and haplotypes GCG, ACG and GCC were positively (all p <0.0001) associated with RA (Table 4).

**Table 4:** *VEGF* haplotypes in rheumatoid arthritis (RA) patients and controls.

Haplotype	RA2n = 1130 (%)	Control 2n = 677 (%)	OR (95% CI)	p*
GAG	193 (16.6)	168 (24.5)	0.615 (0.487 to 0.776)	**<0.0001**
GCG	238 (20.5)	73 (10.6)	2.169 (1.637 to 2.875)	**<0.0001**
AAG	209 (18)	206 (30.1)	0.512 (0.41 to 0.639)	**<0.0001**
ACG	196 (16.9)	43 (6.2)	3.043 (2.155 to 4.296)	**<0.0001**
GCC	101 (8.7)	21 (3)	3.022 (1.87 to 4.884)	**<0.0001**
AAC	157 (13.5)	145 (21.1)	0.584 (0.455 to 0.749)	**<0.0001**
GAC	36 (3.1)	21 (3)	1.014 (0.587 to 1.753)	1

*Fisher’s test. P values in bold face are considered significant.

### Correlation between risk allele of *VEGF* and disease phenotype

In the next step we analysis the possible association of VEGF polymorphisms with severity of RA, in different genetic models (Tables 5–7 and S1–S3 Tables), according to clinical/demographic characteristics of patients. The presence of at last one risk allele was correlated with age of patients, disease duration; selected ACR criteria including DAS-28, Larsen score, HAQ, VAS and RF and anti-CCP presence. Our analysis demonstrated that the number of RA women with -2578 AA genotypes was higher than the number of RA women with -2578 AC or -2578 CC genotypes (Table 6; p = 0.006). We also observed an association of Hemoglobin (Hb) with the -1154 A/G and -634 G/C variants. In both the mean value of Hb was higher in RA patients with polymorphic -1154 GG (Table 5 in) and -634 CC genotypes (Table 7) in comparison to patients with the -1154 A and -634G alleles.

**Table 5:** The disease activity and laboratory parameters in relation to *VEGF*-1154 A/G; recessive model.

**Parameter**	**GG**		**AA+AG**		p*
	***N***	**median (IQR)**	***N***	**median (IQR)**	
Age [years]	*122*	55 (51–63)	*419*	56 (49–65)	0.838
Disease duration [years]	*112*	10 (5–17)	*383*	10 (5–15)	0.213
Larsen	*122*	3 (3–4)	*419*	3 (3–3)	0.272
ESR [mm/h]	*121*	32 (18–50)	*417*	30 (16–48)	0.232
Number of swollen joints	*67*	2 (0–7)	*242*	3 (1–7)	0.152
Number of tender joints	*67*	8 (2–13)	*242*	7 (3–12)	0.565
CRP [mg/L]	*68*	12 (6–30)	*244*	14 (6–34)	0.330
Hemoglobin [g/dL]	*68*	13 (12.1–13.7)	*244*	12.6 (11.5–13.4)	**0.050**
VAS [mm]	*67*	52 (30–75)	*238*	52 (32–69)	0.692
DAS-28	*66*	5.0 (3.8–5.9)	*240*	5.1 (3.9–5.9)	0.850
PLT [x10^3^/mm^3^]	*68*	307 (264.5–367)	*244*	312 (248–383.5)	0.963
Creatinine	*67*	0.7 (0.6–0.8)	*244*	0.7 (0.6–0.8)	0.725
HAQ	*63*	1.6 (0.9–1.9)	*229*	1.5 (1.0–2.0)	0.512
	**GG**		**AA+AG**		p**
	***N***	**n (%)**	***N***	**n (%)**	
Women	*127*	114 (90%)	*431*	378 (88%)	0.527
RF presence	*120*	84 (70%)	*414*	129 (69%)	0.809
anti-CCP presence	*68*	58 (85%)	*246*	196 (80%)	0.297

IQR—interquartile range;

p*—U Mann-Whitney test; p**—χ^2^ test;

p < 0.003 was considered significant (according to Bonferroni correction); P values in bold face are considered significant.

N—number of patients with clinical information

**Table 6:** The disease activity and laboratory parameters in relation to *VEGF*-2578 A/C; dominant model.

**parameter**	**AA**		**AC+CC**		p*
	***N***	**median (IQR)**	***N***	**median (IQR)**	
Age [years]	*162*	55 (49–65)	*379*	57 (51–65)	0.123
Disease duration [years]	*153*	10 (4–17)	*342*	10 (5–15)	0.806
Larsen	*160*	3 (3–3.5)	*381*	3 (3–3)	0.840
ESR [mm/h]	*159*	30 (20–50)	*379*	30 (16–48)	0.257
Number of swollen joints	*89*	3 (1–7)	*220*	3 (1–7)	0.823
Number of tender joints	*89*	7 (4–11)	*220*	8 (2–12.5)	0.942
CRP [mg/L]	*89*	12 (5–34)	*223*	14 (7–31)	0.311
Hemoglobin [g/dL]	*89*	12.8 (11.6–13.6)	*223*	12.6 (11.6–13.5)	0.774
VAS [mm]	*90*	57.5 (32–76)	*215*	51 (30–67)	0.224
DAS-28	*90*	5.0 (4.0–5.9)	*216*	5.1 (3.8–5.9)	0.923
PLT [x10^3^/mm^3^]	*90*	312 (245–383)	*222*	310 (255–383)	0.468
Creatinine	*90*	0.7 (0.6–0.8)	*221*	0.7 (0.6–0.8)	0.732
HAQ	*86*	1.5 (0.8–1.9)	*206*	1.5 (1.0–2.0)	0.357
	**AA**		**AC+CC**		p**
	*N*	n (%)	*N*	n (%)	
Women	*166*	156 (94%)	*392*	336 (86%)	**0.006**
RF presence	*161*	109 (68%)	*373*	260 (70%)	0.646
anti-CCP presence	*90*	71 (79%)	*224*	183 (82%)	0.567

IQR—interquartile range;

p*—U Mann-Whitney test; p**—χ^2^ test;

p < 0.003 was considered significant (according to Bonferroni correction); P values in bold face are considered significant.

N—number of patients with clinical information

**Table 7:** The disease activity and laboratory parameters in relation to *VEGF* -634 G/C; recessive model.

**Parameter**	**CC**		**GG+GC**		p*
	***N***	**median (IQR)**	***N***	**median (IQR)**	
Age [years]	*45*	55 (49–66)	*496*	56 (50–64.5)	0.870
Disease duration [years]	*44*	10 (5–15)	*451*	10 (5–16)	0.876
Larsen	*46*	3 (2–3)	*495*	3 (3–4)	0.638
ESR [mm/h]	*46*	33.5 (20–43)	*492*	30 (16.5–50)	0.542
Number of swollen joints	*25*	4 (2–6)	*284*	3 (1–8)	0.991
Number of tender joints	*25*	9 (4–14)	*284*	7 (3–12)	0.239
CRP [mg/L]	*26*	11.9 (5–21)	*286*	13.5 (6–33)	0.437
Hemoglobin [g/dL]	*26*	13.2 (11.9–14.0)	*286*	12.6 (11.5–13.4)	**0.052**
VAS [mm]	*26*	56.5 (40–70)	*279*	52 (31–70)	0.578
DAS-28	*26*	5.7 (4.2–6.2)	*280*	5.0 (3.9–5.9)	0.277
PLT [x10^3^/mm^3^]	*26*	294 (242–353)	*286*	315 (255–384)	0.437
Creatinine	*26*	0.7 (0.6–0.8)	*285*	0.7 (0.6–0.8)	0.554
HAQ	*26*	1.7 (1.3–2.3)	*266*	1.5 (0.9–2.0)	0.193
	**CC**		**GG+GC**		p**
	***N***	**n (%)**	***N***	**n (%)**	
Women	*46*	42 (91%)	*512*	450 (88%)	0.492
RF presence	*46*	33 (72%)	*488*	336 (69%)	0.686
anti-CCP presence	*26*	24 (92%)	*288*	230 (80%)	0.199

IQR—interquartile range;

p*—U Mann-Whitney test; p**—χ^2^ test;

p < 0.003 was considered significant (according to Bonferroni correction); P values in bold face are considered significant.

N—number of patients with clinical information

### Influence of the *VEGF* SNPs in the risk of CV disease in RA patients

Next, we investigated whether VEGF gene variants was a risk factor for CVD in patients with RA. At genotype level we observed no significant differences in genotype frequencies of all examined VEGF variants between RA patients with and without CVD (S4 Table).

### VEGF protein level in patients/controls and in relation to RA clinical parameters and CVD presence

**Fig 2:**
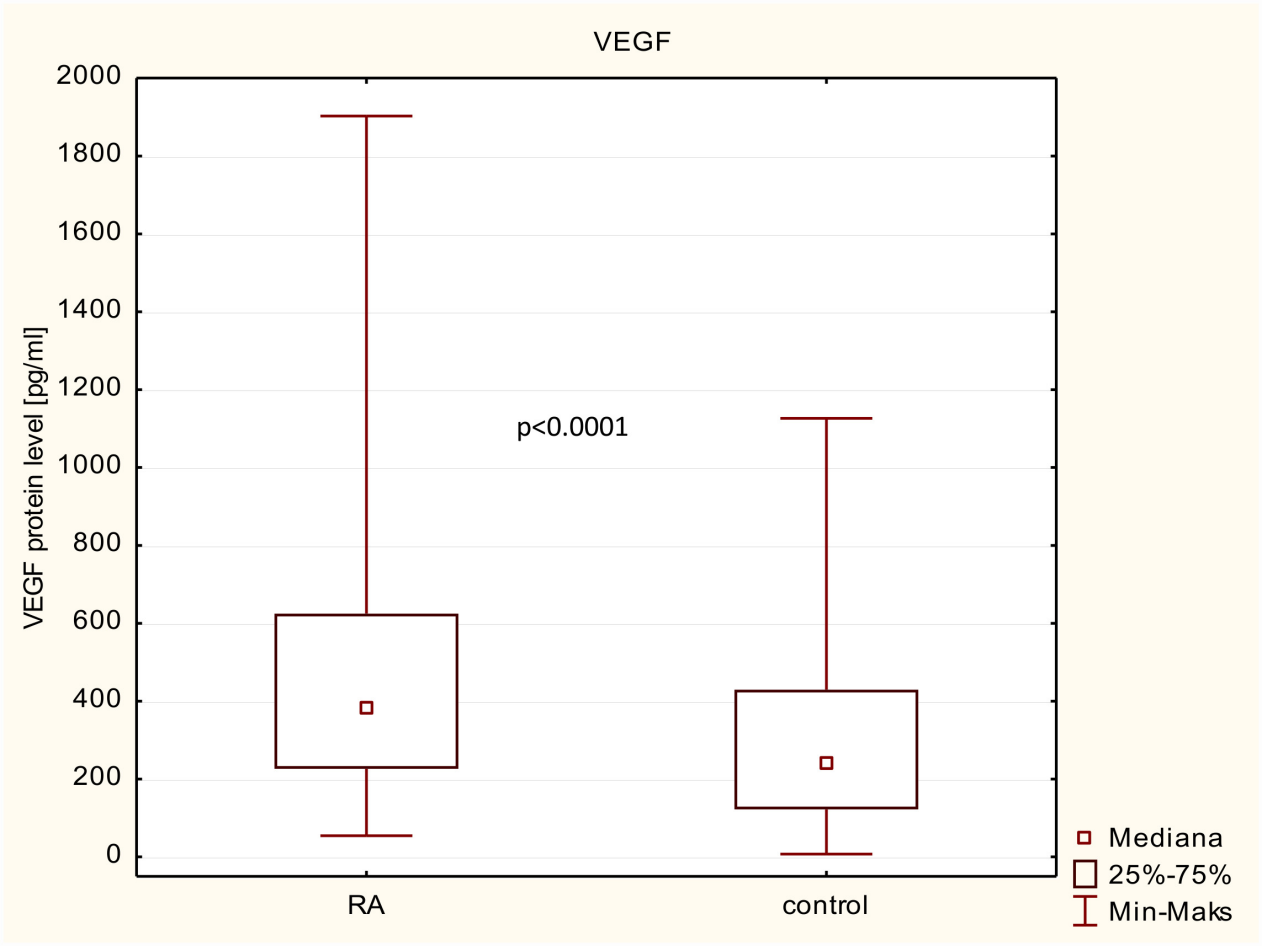
Variation in VEGF expression levels in patients with RA and control group.

We also examined VEGF protein expression levels in serum from 325 RA patients and 293 healthy subjects, recruit from the genetic study cohort. The VEGF protein expression levels were significantly higher in RA patients than in healthy controls (385 vs 239 pg/ml; p<0.0001; Fig 2). We next conducted a comparative analysis between mean value of VEGF protein serum levels and clinical parameters of RA patients (Table 8). Patients were divided into two groups: I group included the RA patients with the higher disease activity as well as RF, anti-CCP and CVD presence; whereas II group included the RA patients with the lowest disease activity without RF, anti-CCP and CV disease. No significant relationship was found between the levels and patient demographics including age, sex, and disease duration. Also, no significant association was observed with autoantibodies presence, clinical parameters of inflammation (DAS-28) and cardiovascular events. Although, we observed that VEGF has shown a tendency to positively correlation with age of RA patients. Patients with age >56 years had a higher VEGF levels compering with those with age <56 years (p = 0.086). Moreover, VEGF showed a highly significant positive correlation with inflammatory marker—CRP. The VEGF levels was higher in RA patients with CRP>13 (median: 445; IQR: 243–676) compared with RA patients with CRP<13 (median322; IQR: 190–527; p = 0.005). We also observed that VEGF serum levels were higher in RA patients with number of swollen joints> 3, ESR ≥ 30, DAS-28 ≥ 5,0 and disease duration≥ 10, however, this association was not significant.

**Table 8:** Correlation of VEGF protein expression level with the various clinical characteristics in RA.

Parameter		VEGF protein level			VEGF protein level		p
	*parameter group I*	*N*	median (IQR)	*parameter group II*	*N*	median (IQR)	
Age	age ≥ 56	*181*	399 (243–674)	age < 56	*145*	340 (203–564)	0.087
sex	women	*299*	375 (224–613)	men	*27*	418 (293–754)	0.190
RF	RF +	*199*	380 (226–635)	RF -	*104*	377 (190–569)	0.428
anti-CCP	a-CCP +	*251*	391 (233–635)	a-CCP -	*59*	323 (177–513)	0.112
disease duration	≥ 10	*150*	389 (206–319)	< 10	*117*	326 (232–519)	0.478
number of tender joints	≥ 7	*166*	339 (214–587)	< 7	*138*	405 (239–627)	0.213
number of swollen joints	≥ 3	*172*	406 (225–661)	< 3	*132*	340 (220–565)	0.155
ESR	≥ 30	*137*	405 (233–648)	< 30	*171*	346 (206–581)	0.311
CRP	≥ 13	*159*	445 (243–676)	< 13	*148*	322 (190–527)	**0.005**
DAS-28	≥ 5,0	*153*	391 (237–635)	< 5,0	*148*	349 (198–584)	0.368
HAQ	≥ 1.5	*157*	340 (209–635)	< 1.5	*131*	393 (245–625)	0.194
cardiovascular diseases	CVD +	*131*	340 (194–625)	CVD -	*174*	397 (239–635)	0.277

p—Mann-Whitney U test or chi-squared test with Yates correction. P values in bold face are considered significant. N: no. patients with clinical information; n: no. patients with positive clinical manifestation; IQR: interquartile range; DAS28: Disease Activity Score for 28 joints; VAS: visual analog scale (range 0–100); HAQ: Health Assessment Questionnaire (range 0–3); CRP: C-reactive protein; ESR: erythrocyte sedimentation rate; PLT: platelet; RF: rheumatoid factor (> 34 IU ⁄ ml); anti-CCP: anticyclic citrullinated peptide antibodies (> 17 U⁄ ml).

### Impact of VEGF polymorphisms on its expression

Finally, we investigated whether VEGF genetic variations had an impact on dysregulation of VEGF at the protein levels.

First, we performed the correlation between VEGF expression levels in RA patients and the healthy subjects in relation to VEGF-1154 A/G (rs1570360), -2578 A/C (rs699947) and -634 G/C (rs2010963) genotypes. In this case, we found no significant association between VEGF genotypes and its serum levels, either among RA patients and/or in healthy subjects (S1 Fig).

**Fig 3:**
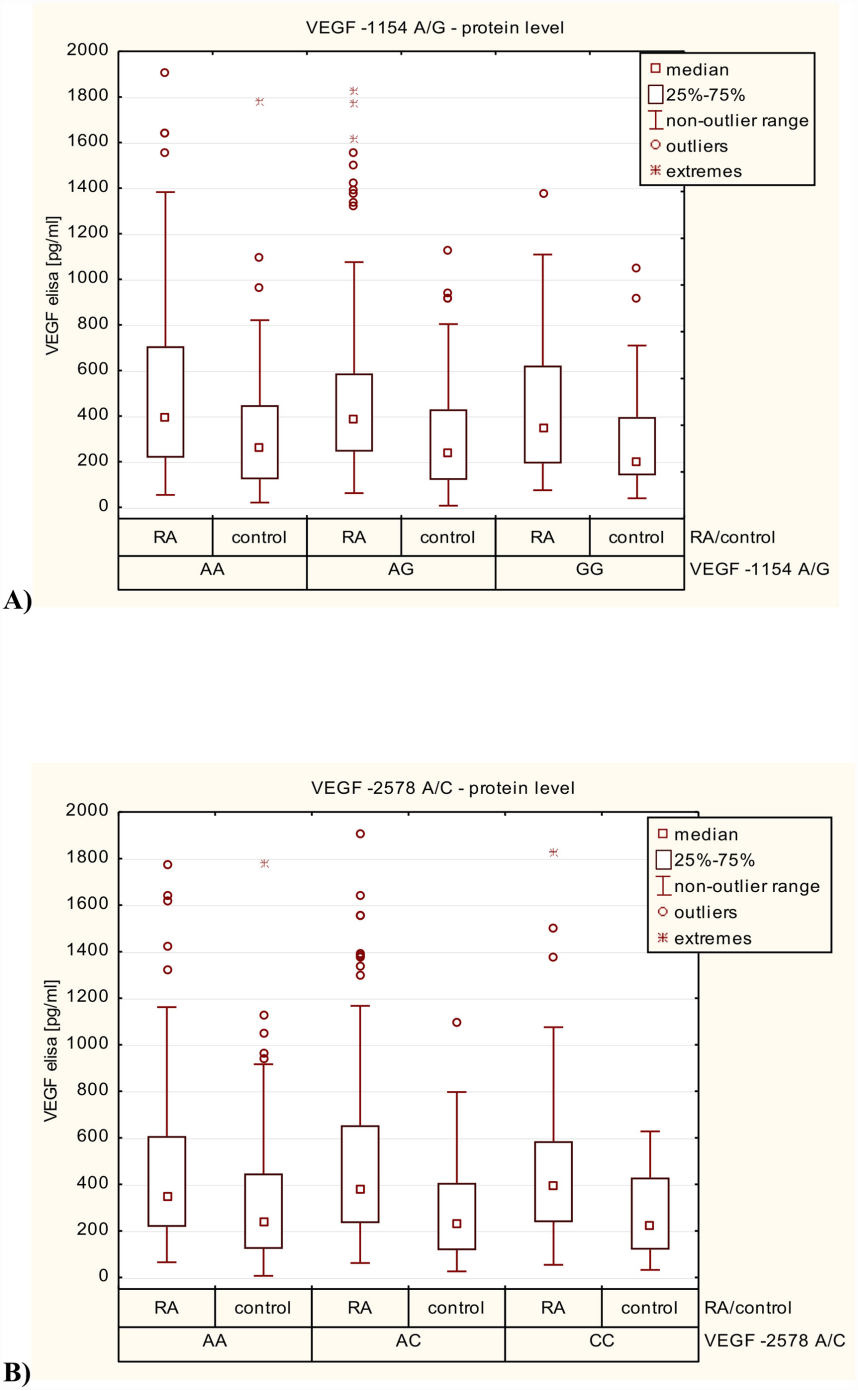
Variation in VEGF expression levels in RA patients and control group in relation to: **A)**—1154 A/G (rs1570360) VEGF genotypes; **B)**—2578 A/C (rs699947) VEGF genotypes; **C)**—634 G/C (rs2010963) VEGF genotypes.

In the nest step, we conducted a comparative analysis between patients and controls according to -1154 A/G (rs1570360), -2578 A/C (rs699947) and -634 G/C (rs2010963) VEGF genotypes. VEGF serum levels in RA patients with -1154 AA, AG and GG genotypes were significantly higher than controls with the same genotypes (Fig 3A). Increased serum levels of VEGF was also observed in RA patients with -2578 AA, AC and CC genotypes compared to healthy subjects (Fig 3B). In RA patients with -634 GG and GC genotypes the VEGF levels was higher than in controls with the same genotypes. Only, serum levels of VEGF among patients with RA who had the– 634 CC genotype were not significantly different from those detected in the sera of healthy donors with –634 CC genotypes (Fig 3C).

## Discussion

In this study we analyzed SNPs in the *VEGF* gene at position -1154 A/G, -2578 A/C and -634 G/C and identified two SNPs, which are strongly associated with susceptibility of RA. These findings indicated that VEGF may be involved in the RA pathogenesis and confirm previously association between genetic variants in VEGF, serum level of VEGF protein and RA [2, 5, 12, 22, 25–28]. To the best our knowledge this is the first report showing the positive association between VEGF gene polymorphisms and susceptibility to RA. In this study, we presented that the minor allele of the two polymorphisms at position -1154 A/G and -2578 A/C, located in the 5’-flanking region of the VEGF gene, may be a genetic risk factor for RA in the Polish population. Moreover, the RA patients who carried the haplotype GCG or GCC of -1154/-2578/-634 were more susceptible to RA, suggesting that the effect of the gene on disease risk may not be limited to a single SNP. In contrast, a study on a Korean [5, 13], England [22], Spanish [12, 25] and China [2] populations found no association between the VEGF -1154 A/G and -2578 A/C polymorphisms and RA. Studies in different ethnic groups may have submitted differing results.

The endothelial cell activation marker VEGF is an excellent candidate for the monitoring of disease activity, erosive progression and treatment response in rheumatoid arthritis patients [14, 16, 29]. The most important factor leading to chronic RA as well as deformity development is the pannus formation [30–32]. It is suggested that VEGF play a central role in the pannus formation and joint destruction, not only by angiogenesis but also by the enhancement of inflammation through the recruiting monocytes to the synovium [16]. Angiogenesis, which is involved in the regulation of several soluble and cell surface-bound factors, play a central role in the RA pathogenesis for a long time [11]. Researchers have tried to find an answer on the question of which came first in RA: angiogenesis or chronic inflammation, but now we know the both processes are closely interrelated [11]. Moreover, inhibition of angiogenesis, which in animals models of arthritis leads to attenuation of severity of the arthritis, has been discussed as a therapeutic target in the arthritis and new intervention in RA [11, 16].

Base on the above observations, we attempt to find the correlation between *VEGF* gene polymorphisms and the clinical phenotype of rheumatoid arthritis. In this study, the polymorphic -2578 C allele, that is a risk allele for development of RA in our population, has been presented less frequently in women than wild type -2578 A allele. Moreover, we also observed that polymorphic -1154 GG and -634 CC genotypes have shown an association with mean value of hemoglobin, which was higher in RA patients with those genotypes. However, in our study we demonstrated no correlation between of the *VEGF* genetic variants and cardiovascular events in our RA patients. The results demonstrated here are in agreement with some studies [12, 25], but not consistent with other [22, 33] in respect to genetic variants located within of the VEGF gene. Discrepancies between reports may be explained by the heterogeneity of the rheumatoid arthritis, ethnicity as well as sample size under study. Although, our sample size may be a limitation of this study and it may not be large enough to detect an association of a gene polymorphisms with the some effect of RA; however, in contrast to other studies, our cohort has a homogenous ancestry with detailed clinical data. Additional studies with larger numbers are needed to validate the association. Moreover, commonly occurring alleles may have low penetrance and only the combined effects of susceptibility genes as well as gene-environmental interactions may interfere the true association between polymorphism and susceptibility/severity of disease.

In this study we also determine the expression of VEGF protein associated with RA. We demonstrated that VEGF protein levels in serum from RA patients were significantly higher than those in healthy subjects, reflecting the angiogenesis and/or chronic inflammation in patients with RA and attempt to keep it under control. Moreover, we also found that serum VEGF levels were significantly increased in an older group of patients as well as correlated with inflammatory marker such as CRP, which was consistent with the previous reports [11, 34]. CRP is the most commonly used biochemical markers as an index of disease activity. In comparison to other studies [21, 29, 34–40], we also observed that VEGF serum levels were higher in RA patients with number of swollen joints > 3, ESR ≥ 30, DAS-28≥ 5,0 and disease duration≥ 10, however, this association was not significant. Based on these observations, serum VEGF levels may serve as a potential biomarker in monitoring of the disease activity and joint destruction. Overall, our data are in agreement with previous reports and demonstrating no relationship between VEGF serum levels and CVD events in our rheumatoid arthritis patients [11]. Furthermore, while the -2578 A/C and -1154 A/G polymorphisms have been previously shown to affect VEGF serum levels [22, 41, 42], the result presented here and elsewhere [43–45] do not support an influence of both these genetic variants on VEGF expression level.

In conclusion, our study provides evidence that VEGF -1154 A/G and -2578 A/C genetic variants may be a genetic susceptibility factor for RA and that VEGF serum levels increased in RA patients with higher disease activity. High VEGF expression may lead to the unnatural stimulation of T cells, macrophages and endothelial cells for the production of proinflammatory cytokines as well as to form new blood vessels, implicating the immune response and angiogenesis in the pathogenesis of RA.

## Supporting Information

S1 Fig Comparison of serum VEGF levels among VEGF genotypes in RA patients (A-C) and in control group (D-F). (DOC)

S1 Table The disease activity and laboratory parameters in relation to *VEGF* -1154 A/G; dominant model. (DOC)

S2 Table The disease activity and laboratory parameters in relation to VEGF -634 G/C; dominant model. (DOC)

S3 Table The disease activity and laboratory parameters in relation to VEGF -2578 A/C; recessive model. (DOC)

S4 Table Distribution of genotypes and allele frequencies of VEGF SNPs among RA patients with CVD and without CVD. (DOC)
