# Supplementary material for: Relationship between VEGF Gene Polymorphisms and Serum VEGF Protein Levels in Patients with Rheumatoid Arthritis
Source: PLoS One. 2016 Aug 11;11(8):e0160769. doi: 10.1371/journal.pone.0160769 (PMC4981324; doi:10.1371/journal.pone.0160769)
Supplement: S1 Fig — (DOC) [file pone.0160769.s001.doc]

**A) p = 0.697**

**B)** p = 0.889

**C)** p = 0.636

**D) p = 0.631**

**E)** p = 0.840

**F)** p = 0.637

**Figure S1.** Comparison of serum VEGF levels among VEGF genotypes in RA patients (A-C) and in control group (D-F).
