# Supplementary material for: Relationship between VEGF Gene Polymorphisms and Serum VEGF Protein Levels in Patients with Rheumatoid Arthritis
Source: PLoS One. 2016 Aug 11;11(8):e0160769. doi: 10.1371/journal.pone.0160769 (PMC4981324; doi:10.1371/journal.pone.0160769)
Supplement: S1 Table — (DOC) [file pone.0160769.s002.doc]

**Table S1:** The disease activity and laboratory parameters in relation to *VEGF* -1154 A/G; dominant model

| **Parameter** | **AA** | | **AG+GG** | | **p*** |
| --- | --- | --- | --- | --- | --- |
|  | ***N*** | **median (IQR)** | ***N*** | **median (IQR)** |  |
| Age [years] | *128* | 58 (51 – 65.5) | *413* | 55 (49 – 64) | 0.102 |
| Disease duration [years] | *116* | 10 (5 – 16) | *379* | 10 (5 – 15) | 0.941 |
| Larsen | *127* | 3 (3 – 3) | *414* | 3 (3 – 4) | 0.215 |
| ESR [mm/h] | *127* | 31 (18 – 48) | *411* | 29 (16 – 50) | 0.751 |
| Number of swollen joints | *68* | 3 (1 – 7) | *241* | 3 (1 – 7) | 0.368 |
| Number of tender joints | *68* | 7 (4 – 11.5) | *241* | 8 (3 – 12) | 0.898 |
| CRP [mg/L] | *70* | 15 (5 – 41) | *242* | 13 (6 – 30) | 0.425 |
| Hemoglobin [g/dL] | *70* | 12.5 (11.5 – 13.1) | *242* | 12.8 (11.6 – 13.6) | 0.310 |
| VAS [mm] | *68* | 53 (31.5 – 68) | *237* | 52 (32 – 70) | 0.789 |
| DAS-28 | *68* | 5.1 (4.3 – 5.8) | *238* | 5.0 (3.8 – 5.9) | 0.507 |
| PLT [x10^3^/mm^3^] | *70* | 319 (256 – 383) | *242* | 308 (252 – 381) | 0.696 |
| Creatinine | *69* | 0.7 (0.6 – 0.8) | *242* | 0.7 (0.6 – 0.8) | 0.365 |
| HAQ | *68* | 1.5 (1.0 – 2.1) | *224* | 1.5 (0.9 – 2.0) | 0.557 |
|  | **AA** | | **AG+GG** | | **p**** |
|  | ***N*** | **n (%)** | ***N*** | **n (%)** |  |
| Women | *132* | 111 (84 %) | *426* | 381 (89 %) | 0.098 |
| RF presence | *127* | 91 (72 %) | *407* | 278 (68 %) | 0.476 |
| anti-CCP presence | *70* | 55 (79 %) | *244* | 199 (82 %) | 0.575 |

IQR – interquartile range;

p* - U Mann-Whitney test; p** - χ^2^ test;

p < 0.003 was considered significant (according to Bonferroni correction);

N – number of patients with clinical information
