# Supplementary material for: Relationship between VEGF Gene Polymorphisms and Serum VEGF Protein Levels in Patients with Rheumatoid Arthritis
Source: PLoS One. 2016 Aug 11;11(8):e0160769. doi: 10.1371/journal.pone.0160769 (PMC4981324; doi:10.1371/journal.pone.0160769)
Supplement: S2 Table — (DOC) [file pone.0160769.s003.doc]

**Table S2: The disease activity and laboratory parameters in relation to *VEGF* -634 G/C; dominant model**

| **Parameter** | **GG** | | **GC+CC** | | **p*** |
| --- | --- | --- | --- | --- | --- |
|  | ***N*** | **median (IQR)** | ***N*** | **median (IQR)** |  |
| Age [years] | *273* | 57 (50 – 64) | *268* | 55.5 (49 – 65) | 0.887 |
| Disease duration [years] | *244* | 10 (5 – 15.5) | *251* | 10 (5 – 16) | 0.925 |
| Larsen | *274* | 3 (3 – 3) | *267* | 3 (3 – 4) | 0.816 |
| ESR [mm/h] | *271* | 29 (16 – 50) | *267* | 30 (19 – 48) | 0.501 |
| Number of swollen joints | *153* | 3 (1 – 7) | *156* | 3 (1 – 7.5) | 0.642 |
| Number of tender joints | *153* | 7 (2 – 12) | *156* | 8 (4 – 12) | 0.281 |
| CRP [mg/L] | *155* | 12.9 (6 – 31) | *157* | 14 (6 – 34) | 0.790 |
| Hemoglobin [g/dL] | *155* | 12.8 (11.6 – 13.6) | *157* | 12.5 (11.6 – 13.5) | 0.827 |
| VAS [mm] | *150* | 52 (33 – 71) | *155* | 52 (30 – 69) | 0.468 |
| DAS-28 | *150* | 5.1 (3.8 – 5.8) | *156* | 5.0 (3.9 – 6.0) | 0.605 |
| PLT [x10^3^/mm^3^] | *154* | 310 (243 – 385) | *158* | 312 (257 – 381) | 0.482 |
| Creatinine | *153* | 0.7 (0.6 – 0.8) | *158* | 0.7 (0.6 – 0.8) | 0.468 |
| HAQ | *148* | 1.5 (0.9 – 2.0) | *144* | 1.5 (1.0 – 2.0) | 0.933 |
|  | **GG** | | **GC+CC** | | **p**** |
|  | ***N*** | **n (%)** | ***N*** | **n (%)** |  |
| Women | *282* | 249 (88 %) | *276* | 243 (88 %) | 0.926 |
| RF presence | *269* | 190 (71 %) | *265* | 179 (68 %) | 0.441 |
| anti-CCP presence | *157* | 123 (78 %) | *157* | 131 (83 %) | 0.251 |

IQR – interquartile range;

p* - U Mann-Whitney test; p** - χ^2^ test;

p < 0.003 was considered significant (according to Bonferroni correction);

N – number of patients with clinical information
