# Supplementary material for: Relationship between VEGF Gene Polymorphisms and Serum VEGF Protein Levels in Patients with Rheumatoid Arthritis
Source: PLoS One. 2016 Aug 11;11(8):e0160769. doi: 10.1371/journal.pone.0160769 (PMC4981324; doi:10.1371/journal.pone.0160769)
Supplement: S3 Table — (DOC) [file pone.0160769.s004.doc]

**Table S3: The disease activity and laboratory parameters in relation to *VEGF* -2578 A/C; recessive model**

| **Parameter** | **CC** | | **AA+AC** | | **p*** |
| --- | --- | --- | --- | --- | --- |
|  | ***N*** | **median (IQR)** | ***N*** | **median (IQR)** |  |
| Age [years] | *140* | 56 (50 – 64) | *401* | 56 (50 – 65) | 0.729 |
| Disease duration [years] | *127* | 9 (5 – 13) | *368* | 10 (5 – 16) | 0.271 |
| Larsen | *140* | 3 (3 – 4) | *401* | 3 (3 – 3) | 0.270 |
| ESR [mm/h] | *141* | 30 (15 – 50) | *397* | 30 (18 – 50) | 0.687 |
| Number of swollen joints | *80* | 2.5 (0 – 7) | *229* | 3 (1 – 8) | 0.268 |
| Number of tender joints | *80* | 7 (2 – 12) | *229* | 8 (4 – 12) | 0.311 |
| CRP [mg/L] | *83* | 12.2 (6 – 30.4) | *229* | 13.8 (6 – 32) | 0.728 |
| Hemoglobin [g/dL] | *82* | 12.5 (11.5 – 13.1) | *230* | 12.7 (11.6 – 13.6) | 0.313 |
| VAS [mm] | *77* | 51 (30 – 72) | *228* | 52 (32 – 69) | 0.599 |
| DAS-28 | *78* | 4.9 (3.5 – 6.0) | *228* | 5.1 (4.0 – 5.9) | 0.598 |
| PLT [x10^3^/mm^3^] | *82* | 298.5 (254 – 392) | *230* | 317 (254 – 381) | 0.874 |
| Creatinine | *81* | 0.7 (0.6 – 0.8) | *230* | 0.7 (0.6 – 0.8) | 0.966 |
| HAQ | *75* | 1.6 (1.0 – 2.1) | *217* | 1.5 (0.9 – 2.0) | 0.444 |
|  | **CC** | | **AA+AC** | | **p**** |
|  | ***N*** | **n (%)** | ***N*** | **n (%)** |  |
| Women | *144* | 123 (85 %) | *414* | 369 (89 %) | 0.235 |
| RF presence | *139* | 90 (65 %) | *395* | 279 (71 %) | 0.197 |
| anti-CCP presence | *83* | 66 (80 %) | *231* | 188 (81 %) | 0.711 |

IQR – interquartile range;

p* - U Mann-Whitney test; p** - χ^2^ test;

p < 0.003 was considered significant (according to Bonferroni correction);

N – number of patients with clinical information
