# Supplementary material for: Relationship between VEGF Gene Polymorphisms and Serum VEGF Protein Levels in Patients with Rheumatoid Arthritis
Source: PLoS One. 2016 Aug 11;11(8):e0160769. doi: 10.1371/journal.pone.0160769 (PMC4981324; doi:10.1371/journal.pone.0160769)
Supplement: S4 Table — (DOC) [file pone.0160769.s005.doc]

**Table S4.** Distribution of genotypes and allele frequencies of VEGF SNPs among RA patients with CVD and without CVD.

| **genotype** | **patients with CVD**  **N=134** | **patients without CVD**  **N=177** | **p** |
| --- | --- | --- | --- |
|  | **n (%)** | **n (%)** |  |
| **VEGF-1154 (G/A)** | | | 0.174 |
| AA | 34 (25%) | 36 (20%) |  |
| AG | 66 (49 %) | 106 (60 %) |  |
| GG | 34 (25 %) | 35 (20 %) |  |
| **VEGF -2578 (C/A)** | | | 0.815 |
| AA | 40 (30 %) | 49 (28 %) |  |
| AC | 61 (45 %) | 79 (45 %) |  |
| CC | 33 (25 %) | 49 (28 %) |  |
| **VEGF -634 (G\C)** | | | 0.547 |
| GG | 70 (52 %) | 95 (54 %) |  |
| GC | 53 (40 %) | 72 (41 %) |  |
| CC | 11 (8 %) | 9 (5 %) |  |

p - χ2 test with Yate’ correction, p≤0,05 was considered as significant
